# Role of Endocrine-Disrupting Chemicals in the Pathogenesis of Non-Alcoholic Fatty Liver Disease: A Comprehensive Review

**DOI:** 10.3390/ijms22094807

**Published:** 2021-05-01

**Authors:** Raquel Cano, José L. Pérez, Lissé Angarita Dávila, Ángel Ortega, Yosselin Gómez, Nereida Josefina Valero-Cedeño, Heliana Parra, Alexander Manzano, Teresa Isabel Véliz Castro, María P. Díaz Albornoz, Gabriel Cano, Joselyn Rojas-Quintero, Maricarmen Chacín, Valmore Bermúdez

**Affiliations:** 1 Endocrine and Metabolic Diseases Research Center, School of Medicine, University of Zulia, Maracaibo 4004, Venezuela; raquelamiracano@gmail.com (R.C.); joseluispv2811@gmail.com (J.L.P.); angelort94@hotmail.com (Á.O.); yoselin_gomezgil@hotmail.com (Y.G.); helianapp20@hotmail.com (H.P.); amanzano_8@hotmail.com (A.M.); mariadiazalbornoz@hotmail.com (M.P.D.A.); 2 Escuela de Nutrición y Dietética, Facultad de Medicina, Universidad Andres Bello, Sede Concepción 4260000, Chile; lisse.angarita@unab.cl; 3 Carrera de Laboratorio Clínico, Facultad de Ciencias de la Salud, Universidad Estatal del Sur de Manabí, Jipijapa E482, Ecuador; nereida.valero@unesum.edu.ec (N.J.V.-C.); teresa.veliz@unesum.edu.ec (T.I.V.C.); 4 Insitute für Pharmazie, Freie Universitänt Berlin, Königin-Louise-Strabe 2-4, 14195 Berlin, Germany; gabriel.simon.cano@fu-berlin.de; 5 Division of Pulmonary and Critical Care Medicine, Brigham and Women’s Hospital, Harvard Medical School, Boston, MA 02115, USA; jrojasquintero@bwh.harvard.edu; 6 Facultad de Ciencias de la Salud. Barranquilla, Universidad Simón Bolívar, Barranquilla 55-132, Colombia; m.chacin@unisimonbolivar.edu.co

**Keywords:** non-alcoholic fatty liver disease, endocrine-disrupting chemicals, liver disorder, environmental pollutants, exposure

## Abstract

Non-alcoholic fatty liver disease (NAFLD) is considered the most common liver disorder, affecting around 25% of the population worldwide. It is a complex disease spectrum, closely linked with other conditions such as obesity, insulin resistance, type 2 diabetes mellitus, and metabolic syndrome, which may increase liver-related mortality. In light of this, numerous efforts have been carried out in recent years in order to clarify its pathogenesis and create new prevention strategies. Currently, the essential role of environmental pollutants in NAFLD development is recognized. Particularly, endocrine-disrupting chemicals (EDCs) have a notable influence. EDCs can be classified as natural (phytoestrogens, genistein, and coumestrol) or synthetic, and the latter ones can be further subdivided into industrial (dioxins, polychlorinated biphenyls, and alkylphenols), agricultural (pesticides, insecticides, herbicides, and fungicides), residential (phthalates, polybrominated biphenyls, and bisphenol A), and pharmaceutical (parabens). Several experimental models have proposed a mechanism involving this group of substances with the disruption of hepatic metabolism, which promotes NAFLD. These include an imbalance between lipid influx/efflux in the liver, mitochondrial dysfunction, liver inflammation, and epigenetic reprogramming. It can be concluded that exposure to EDCs might play a crucial role in NAFLD initiation and evolution. However, further investigations supporting these effects in humans are required.

## 1. Introduction

Non-alcoholic fatty liver disease (NAFLD) encompasses a broad spectrum of pathologies, ranging from simple steatosis (NAFL) to non-alcoholic steatohepatitis (NASH), with potential progression to cirrhosis and hepatocellular carcinoma (HCC), in individuals without significant alcohol consumption [1,2]. This pathology is considered a manifestation of the metabolic syndrome (MetS) due to its association with obesity, insulin resistance (IR), and type 2 diabetes mellitus (T2DM) [3,4]. It is a major health problem, representing the leading cause of chronic liver disease worldwide [3]. The global prevalence of NAFLD is around 25% in adults, with notable variability among continents, the highest rates belonging to South America (31%) and the Middle East (32%), followed by Asia (27%), North America (24%), Europe (23%), and Africa (14%) as the least affected area [5,6].

During recent decades, mechanistic advances have emerged to explain NAFLD development. The currently accepted theory is the “multiple-hit model”, which involves the interaction of genetic, dietary, and environmental factors as mediators in the etiopathogenesis [7]. These factors promote the onset of IR in adipose tissue, lipolysis and consequent adipocyte dysfunction, thus increasing influx of free fatty acids (FFAs) into the liver [8,9]. This increase in FFA levels translates into the re-esterification and accumulation of triglycerides (TG) in the liver, simultaneously with the accumulation of other lipid metabolites, such as diacyl-glycerols, long-chain acylcarnitines, and ceramides. These lipotoxic intermediates are responsible for harmful effects such as mitochondrial dysfunction, oxidative stress, and chronic liver inflammation involved in disease progression [8,10,11].

In this context, it is necessary to mention environmental pollutants. These molecules have acquired significant relevance as a consequence of industrialization growth due to their potential involvement in the development of multiple pathologies [12]. Amid environmental pollutants, endocrine-disrupting chemicals (EDCs) are a heterogeneous group of substances that include synthetic products used as solvents/lubricants, plastics, plasticizers, pesticides, fungicides, pharmaceutical agents, and natural products present in human and animal foods [13,14,15]. These substances are acknowledged as disruptors, able to interfere with hormonal signaling and, consequently, cause hormonal dysregulation that mediates various metabolic disorders. Several experimental investigations have shown that acute or chronic exposure to EDCs contributes significantly to NAFLD development [15,16,17]. Therefore, this review aims to summarize the effects of exposure to EDCs on the initiation and progression of NAFLD from a molecular and clinical perspective.

## 2. Endocrine-Disrupting Chemicals: An Overview

Although this research field has gained particular interest in recent years, knowledge of the potential role of chemicals on hormonal function dates back to the mid-20th century [18]. In 1962, the book “Silent Springs” by Rachel Carson highlighted the long-term consequences of using herbicide and fungicide products on wildlife [19]. Afterward, in 1991, at the Wingspread meeting in Wisconsin, the term “endocrine disruptor” was introduced [20]. Likewise, various international entities have defined these agents. According to the US Environmental Protection Agency (EPA), an EDC is an exogenous agent that interferes with the production, release, transport, metabolism, binding, action, or elimination of natural hormones in the body responsible for the maintenance of homeostasis and the regulation of developmental processes.

Furthermore, the European Commission highlighted three characteristics that EDCs should exhibit: (I) endocrine activity, (II) mediation of a deleterious or pathological endocrine mediated-activity, or both, and (III) a cause–effect relationship between the compound and endocrine activity in exposed subjects [21,22]. In the context of these definitions, multiple studies have been carried out to clarify the mechanisms by which EDCs affect hormonal action. The first studies show the estrogenic effects of chemical agents known as xenoestrogens [23,24].

It is well known that EDCs are involved in the signaling of multiple hormones through their activities as nuclear receptor (NR) ligands, exerting agonist or antagonistic actions [25]. Moreover, EDCs could have a potential role in the regulation of genomic expression, promoting epigenetic modifications that result in the development of a wide variety of pathologies by mediating carcinogenic, neurotoxic, hepatotoxic, nephrotoxic, and immunotoxic effects, among others [24,26,27]. Suggestive evidence indicates the existence of more complex processes such as EDC interaction with non-steroidal receptors, transcriptional coactivators, and even enzymatic pathways responsible for endogenous steroid biosynthesis and metabolism [28,29]. At present, there are many manufactured chemicals, of which around 1000 have been identified as EDCs [30].

According to their origin, they can be classified as natural (phytoestrogens, genistein, and coumestrol) or synthetic, and the latter ones can be further subdivided into industrial (dioxins, polychlorinated biphenyls, and alkylphenols), agricultural (pesticides, insecticides, herbicides, and fungicides), residential (phthalates, polybrominated biphenyls, and bisphenol A), and pharmaceutical (parabens). In addition, some heavy metals such as cadmium (Cd), arsenic, and mercury, among others, stand out for their potential as EDCs (Table 1) [31,32]. The exposure pathways to these agents can be variable, with food intake, inhalation of combustion particles, and direct dermal contact being the most common [13,33]. As a result, it is necessary to describe some of the most characterized EDCs to date.

First of all, bisphenol A (BPA) is an EDC classified by the EPA as the third most important pollutant. This molecule, categorized as a weak xenoestrogen, is used in the manufacture of polycarbonate plastics and epoxy resins, which, due to its proximity to food, tends to seep out of the plastic and cause harmful effects. However, its lipophilicity is low and it degrades rapidly, having a half-life of 4 to 5 h [34,35]. Similarly, phthalates are used as plasticizers in coatings, cosmetics, medical tubes, and toys, representing another substance with frequent exposure whose toxicity depends on oxidative stress [36,37,38]. In turn, nonylphenols (NP), also called alkylphenols, are chemicals present as lubricants in oil additives, laundry detergents, emulsifiers, and solubilizers and are responsible for causing widespread contamination in soil, sediments, water, and food. The aforementioned compounds have particular importance for their estrogen-like activity, exerting a competitive inhibition against natural estrogens [39,40].

Under other conditions, regular contact with home products, such as pesticides, represents one of the major sources of contamination, with the particularity that these agents can persist for a long time in the environment [41]. These properties allow pesticides to be part of a group of chemical substances called “persistent organic pollutants” (POPs), which, as a result of their relatively slow degradation rate and high lipophilicity, have an alarming toxic profile, remaining in tissues for prolonged amounts of time, especially in adipose tissue [42,43]. Besides, POPs are semi-volatile, and as a consequence, they can be spread over long distances and are widely dispersed by air and ocean currents. The Stockholm convention designated 12 compounds as the “dirty dozen”. This group includes pesticides, industrial chemicals, and by-products such as aldrin, chlordane, toxaphene, mirex, hexachlorobenzene, heptachlor, furans, endrin, dioxins, dieldrin, dichlorodiphenyltrichloroethane (DDT), and polychlorinated biphenyls (PCBs) [44,45].

Not least, heavy metals constantly pollute the environment when released from industrial and agricultural products. In addition to these, tobacco is also an important release source of these agents. Evidence indicates that organs such as the kidney, liver, and testicles are the most vulnerable to these compounds. Moreover, according to the International Agency for Research on Cancer, arsenic is listed among the 121 Group I carcinogenic agents [46,47,48].

On the other hand, polycyclic aromatic hydrocarbon (PAH) generated by open combustion and natural filtration of oil or coal deposits, or by incomplete combustion of coal, oil, gas, wood, garbage, and tobacco, can contaminate by means of its binding to particles in the air as well as to dishes cooked or roasted at high temperatures [49,50]. Finally, perfluorinated chemicals (PFCs), used primarily in the manufacturing of non-stick cookware, sofas, clothing, waterproof mattresses, food packaging, and fire-fighting materials, are ubiquitous pollutants in the domestic environment. Due to their delayed elimination from the body, with a half-life of approximately 2 to 8 years, they are also classified as POPs. Studies indicate deleterious effects of these compounds on the liver, immune system, and growth [51].

## 3. Endocrine-Disrupting Chemicals Exposure and Liver

The liver, with its multifaceted profile, is one of the first lines of defense against harmful substances, encompassing mechanisms that involve filtration, oxidation, and conjugation of chemical compounds. Enzymatic systems such as cytochrome P450 (CYP) and UDP-glucuronosyltransferase (UGT) allow clearance of more than 90% of these substances [52,53]. Consequently, the liver constitutes the center of xenobiotic metabolism, and its continuous exposure to numerous toxic substances may alter its function [54].

The metabolism of toxins and the mechanisms of liver disruption by EDCs have not yet been fully elucidated. It is plausible to consider that their arrival to the liver corresponds to food intake. Thus, absorption and transport of EDCs are shared with diet lipids through portal circulation [55,56]. On the other hand, these compounds are characterized by being highly lipophilic, a property that allows them to diffuse through cell membranes with great ease, accelerating access to their action site [57,58]. Furthermore, the liver expresses a vast number of NRs such as peroxisome proliferator-activated receptors (PPARs), forming a dimer with retinoid X receptor (RXR), liver X receptor (LXR), aryl hydrocarbon receptor (AhR), constitutive androstane receptors (CAR), and pregnane X receptor (PXR). This places the liver as a critical target for EDCs, requiring further study [59,60].

Once in the liver, activation of NRs by EDCs triggers alteration of hormonal signaling pathways, combined with modulation of the CYP system [59]. This enzymatic system includes numerous isoforms; however, those involved in toxins’ metabolism are CYP1A2, CYP2C9, CYP2C19, CYP2D6, CYP2E1, and CYP3A [61]. The effect of EDCs on this detoxifying system turns out to be controversial. First, it has been established that these compounds tend to inhibit CYP system activity. Studies demonstrate that pesticides, parabens, phthalates, and BPA can reduce the catalytic efficiency of CYP450 [62,63,64]. Similarly, oral administration of NP reduces *cyp2c* expression and *cyp1a1* in vitro activity in rat liver microsomes. From such evidence, it is suggested that altered metabolism together with prolonged exposure are vital factors that favor bioaccumulation of these substances [65,66,67]. On the other hand, this complex can target some EDCs, promoting their transformation into more active metabolites. Such is the case of low molecular weight phthalates such as di-(2-ethylhexyl) phthalate (DEPH), dimethyl-phthalate (DMP), and dibutyl phthalate (DBP), which can undergo phase 1 biotransformation to become monoester hydrolytic metabolites with higher activity [68,69].

The bioaccumulation profile of these substances varies. In particular, BPA, phthalates, and parabens are usually rapidly metabolized and excreted in feces and urine. In contrast, organochlorine pesticides, dioxins, and PCBs as POPs accumulate in adipose tissue and are gradually released into the bloodstream [70,71,72]. Interestingly, Nicolucci et al., using Liquid Chromatography-Electrospray Ionization-Mass Spectrometry (LC/ESI-MS/MS), provided insight into the BPA profile in human plasma and urine and its relation to liver health, observing sustained elevated levels of unconjugated BPA in plasma and urine in subjects with liver disease. These elevated levels suggest impaired first-step metabolism and potential bioaccumulation of BPA in adipose tissue after reaching an exposure threshold. In this sense, the continuous release of this compound to target organs is possible, despite its short half-life [73].

Finally, an excellent way to measure the effect of EDCs on liver function is through classic markers such as alanine aminotransferase (ALT), aspartate aminotransferase (AST), alkaline phosphatase (ALP), and γ-glutamyl transferase (GGT) [74,75]. It has been documented that exposure to various POPs such as PCBs, octachlorodibenzodioxin (OCDD), and some pesticides is associated with increased bilirubin, ALT, and ALP, suggesting a deleterious effect on liver function with exposure to these pollutants [76]. Recently, Baralić et al. [77] explored the effect of oral exposure to a mixture of DEPH, DBP, and BPA in Wistar rats, observing increases in total bilirubin levels, AST (*p* < 0.05), and ALT (*p* < 0.01) along with an increase in total liver weight. The latter corresponds to a possible hepatocellular injury and biliary obstruction, showing a synergistic effect on liver damage induction from EDCs.

## 4. Mechanisms of Action of Endocrine-Disrupting Chemicals in NAFLD

Numerous investigations have confirmed the role of EDC exposure in the development of metabolic disorders. Hence, a new form of categorizing these molecules under the name of metabolism-disrupting chemicals (MDCs) due to their effects on adipogenesis induction, lipid metabolism, and energy balance alteration has emerged [78]. Several preclinical studies have emphasized the mechanistic links between EDC exposure and NAFLD [17,79,80,81]. For instance, Al-Eryani et al. described the potential association between 123 environmental chemicals and NAFLD development in rodents. Within these, pesticides are the most prevalent and PCBs and dioxins the most potent [81]. The effect of these xenobiotics is mediated by their interaction with NRs and other receptors, such as estrogen receptors (ERα and -β), promoting an alteration of liver metabolism through genomic and non-genomic mechanisms [60,82].

**Figure 1:**
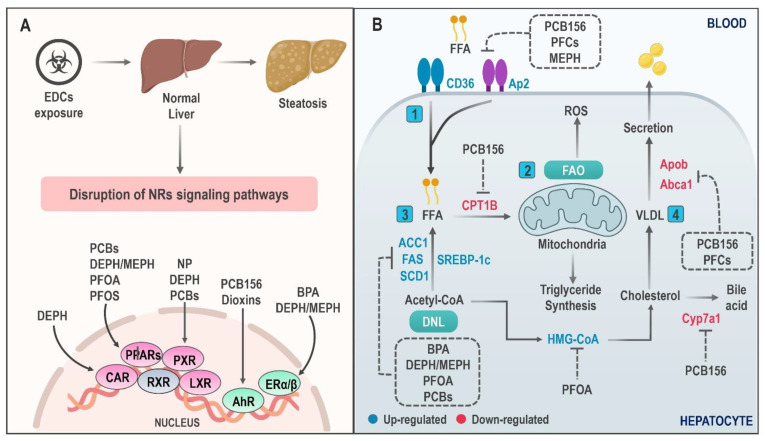
Molecular mechanisms involved in hepatic lipid accumulation induced by EDC exposure. (**A**) The steatosis-inducing effect of EDCs is mediated by binding with different nuclear receptors (NRs). The affinity profile of these substances for their receptors is variable. (**B**) NR signaling disruption with chemical exposure triggers an alteration in lipid metabolic pathways that promote lipids accumulation in the hepatocyte. Key mechanisms of action include (1) significantly increased lipid uptake, (2) decreased fatty acid oxidation, (3) increased expression of key regulators in de novo lipogenesis, and (4) blocking of lipid secretion in the form of VLDL particles and bile acid. Abbreviations: PCB: Polychlorinated Biphenyls; BPA: Bisphenol A; PFOS: Perfluorooctane sulfonate; PFOA: perfluorooctanoic acid; MEPH: Mono-(2-ethylhexyl) phthalate; DEPH: Di-(2-ethylhexyl) phthalate; NP: Nonylphenol; FAO: Fatty acid oxidation; FFA: Free fatty acid; ACC1: Acetyl-CoA carboxylase-1; SCD1: Stearoyl-CoA desaturase-1; FAS: Fatty acid synthase; VLDL: Very low-density lipoprotein; SREBP-1c: Sterol regulatory element-binding protein-1c; CD36: cluster of differentiation 36; DNL: De novo lipogenesis; ROS: reactive oxygen species.

The constitutive activation of these molecular pathways by EDCs leads to the development and progression of NAFLD (Figure 1), triggering a chain of multiple phenomena such as increased hepatic IR, increased accumulation of hepatic TG, mitochondrial dysfunction, and, finally, inflammation and oxidative stress—key in the development of NASH [83].

### 4.1. Hepatic Lipid Accumulation

NAFLD is characterized by an excessive accumulation of hepatic lipids due to an intricate network of events that promote an imbalance between lipid production and elimination. This process is mediated by four main mechanisms: (I) increased lipid uptake in the liver, (II) decreased very low-density lipoprotein (VLDL) particle lipid export, (III) decreased fatty acid oxidation (FAO), and (IV) increased de novo lipogenesis (DNL) [84,85]. This process arises in response to various triggers such as hormonal environment, genetics, drugs, viruses, and, particularly, environmental pollutants [86].

Some EDCs have been documented to directly induce pathological fat aggregation, while others indirectly produce IR and, thus, increase carbohydrates’ conversion to TG in the liver [87]. PCBs are one of the chemicals associated with this fact, and this relationship exists for both dioxin-like (DL) and non-dioxin-like (NDL) groups [88]. Specifically, 2,3,3′,4,4′,5-Hexachlorobiphenyl (PCB156), a DL compound, exerts a multimodal effect on lipids accumulation by acting on PPARα/γ and AhR receptors, promoting an increase in cluster of differentiation 36 (CD36) and a decrease in CPT1B expression. These generate a rise in FFA flux to the liver and a reduction in FAO, respectively. Additionally, exposure to PCB156 may compromise cholesterol homeostasis, interfering with its transport by downregulation of *cyp7a1* and *abca1* [89].

On the contrary, some PFCs such as perfluorooctanoic acid (PFOA), perfluorooctane sulfonate (PFOS), perfluorononanoic acid (PFNA), and perfluorohexanesulfonic acid (PFHxS) are capable of mediating lipids accumulation in the liver by a PPARα-independent mechanism, demonstrated through an increase in *cd36* and *vldlr* expression as well as a reduction in *apob* gene expression. The latter is associated with VLDL secretion in PPAR-null mice; this effect is reported mainly for PFNA and PFHxS [90,91]. In this regard, Yan et al. [92] postulated that PFOA promotes liver lipid synthesis by increasing maturation of sterol regulatory element binding protein (SREBP) and its target genes in a dose-dependent manner in Balb/c mice.

Recently, Zhang et al. [93] showed that PFOA causes a pronounced increase in liver lipid volume in amphibians in a dose- and sex-dependent manner. This increase in TG and total cholesterol (TCHO) levels in response to PFOA exposure is associated with the expression of fatty acid synthase (*fas), gpat,* and *hmg-coa* mediated by PPARγ and SREBP2. In summary, more research is required to elucidate the role of PPARs in the expression of lipogenic genes induced by PFOA exposure.

On the other hand, DEPH and its active metabolite mono-(2-ethylhexyl) phthalate (MEPH) have shown a significant effect on the hepatic accumulation of TGs and, subsequently, on exacerbation of high fat diet (HFD) induced NAFLD in rodents [94]. Furthermore, MEPH exposure has been reported to promote abnormal lipid accumulation in BRL-3A hepatocytes by inhibiting Janus kinase 2/Signal transducer and activator of transcription 5 (JAK2/STAT5) signaling. This demonstrates that STAT5 regulation by MEPH constitutes a crucial factor in the activation of enzymes that participate in the synthesis and transport of fatty acids, such as FAS and ap2 [95]. Bai et al. evaluated MEPH effect on lipids accumulation in HepG2 cells, showing an increase in DNL in the initial hours of exposure due to increased expression of limiting enzymes such as FAS, acetyl-CoA carboxylase-1 (ACC1), and stearoyl-CoA desaturase-1 (SCD1). Interestingly, in later stages of exposure, a detriment of DNL was observed, probably mediated by compensatory mechanisms, so it is assumed that MEPH may also contribute to fatty acid transport to the liver [96].

Finally, another EDC able to promote DNL is BPA [97]. This was demonstrated following an increase in insulin production and expression of *fas, acc1,* and *scd1* after exposure to low doses of BPA (50 µg/kg/day) for 28 days in male CD1 mice (Charles River, Les Oncins, France). This effect of BPA on TG and cholesterol esters’ accumulation in the liver is associated with increased expression of the transcription factor *srebp-1c* [98]. Another BPA effect on steatosis induction through modulation of the endocannabinoid system (ECS) has been described in zebrafish and human hepatocytes [99].

### 4.2. Mitochondrial Dysfunction

Mitochondria play a crucial role in NAFLD pathogenesis, which is even being considered by some authors as a mitochondrial disease [100]. Ultrastructural alterations of the hepatic mitochondria in NAFLD occur in response to a poor adaptation of these organelles to increased lipid content. Consequently, there is deterioration in their biogenesis and dynamics, which results in increased lipid production, generation of reactive oxygen species (ROS), lipid peroxidation (LPO), cytokine production, and cell death [101,102]. The mechanisms involved are a decrease in the mitochondrial respiratory chain (MRC) activity due to an increase in proton leakage and, consequently, a decrease in ATP production [103].

In light of this, EDCs may play a potential role in mitochondrial dysfunction, and their link with NAFLD has been proposed. Studies show that EDCs’ effects on mitochondrial bioenergetics, dynamics, biogenesis, and antioxidant capacity are implicated in the development of IR, T2DM, and MetS [104,105]. With this broad view, it has been shown that atrazine [106] and BPA [107] exert direct mitochondrial toxicity in HepG2 cells through the reduction in mainspring genes involved in mitochondrial function such as mitochondrial transcription factor A (*TFAM*) and sirtuin 1 (*SIRT1*) and MRC interruption, which leads to a decrease in oxidative phosphorylation (OXPHOS) and ATP production.

Likewise, perinatal exposure to BPA in Wistar rats contributed to the development of hepatic steatosis in adult offspring rats by progressively decreasing the activity of complexes I and III of the MRC, OXPHOS, the mitochondrial membrane potential (MMP), and ATP production. BPA also induces changes in factors related to the intrinsic pathways of apoptosis such as caspase-3, Bax, and Bcl-2. It should be noted that the impairment of mitochondrial activity was related to IR and obesity onset; thus, mitochondrial dysfunction induced by BPA may favor the development of NAFLD [108]. Similarly, other EDCs such as NP can exert significant effects on MMP loss and apoptosis of rat hepatocytes by promoting an increase in uncoupling protein-2 (UCP2) [109], deregulating Bax/Bcl-2 activity, and triggering mRNA activation of TNF-α, caspase-9, and Fas/FasL [110], thus promoting the development of mitochondrial dysfunction, liver injury, and steatosis.

In contrast, Cd promotes dose-dependent NAFLD development, inflammation, and fibrosis through mitochondrial damage. This damage shows distinguishing features: abnormal mitochondrial morphology, decreased *fao* gene expression, and decreased mitochondrial DNA (mtDNA) copies in mice treated chronically with this metal (20 weeks) [111]. Cd may suppress SIRT1 signaling, a NAD^+^-dependent deacetylase that regulates lipid metabolism by inducing the expression of essential proteins in fatty acid β-oxidation [111,112]. Therefore, SIRT1 activity regulation through agonists could represent a novel approach to stop NAFLD induced by Cd exposure. In this sense, recent studies have reported favorable effects of SIRT1 activation over pathophysiological NAFLD elements such as glycemic [113,114,115] and lipid impairment [116,117], inflammation [118], and oxidative stress [119], among others [112,120,121].

In addition to mitochondrial toxicity, EDCs also mediate antioxidant machinery deterioration that consequently induces oxidative stress. This mechanism is crucial in NASH progression due to the ROS effect on pro-inflammatory cytokine production and apoptosis of hepatocytes by inducing nuclear factor κappa B (NF-κB) [122,123]. In this regard, a decrease in superoxide dismutase (SOD), catalase (CAT), and glutathione peroxidase (GPx) enzymatic activity with glutathione/glutathione disulfide (GSH/GSSH) ratio appears to be a significant importance event. Oxidative stress has been reported to occur after exposure to BPA [124], PFOS [125], PCB156 [126], MEPH [127], and Cd [111], among others, both in vivo and in vitro. Besides, an increase in mitochondrial ROS following EDC exposure also promotes an increase in 4-Hydroxy-nonenal (4-HNE) and malondialdehyde (MDA) (LPO products and oxidative stress markers), which, in turn, lead to cell membrane disturbance and exacerbation of liver damage [126]. All these findings show that mitochondrial damage mediated by these agents constitutes a significant factor in NAFLD development.

### 4.3. Mechanisms of Hepatic Inflammation Mediated by EDCs

Hepatocellular damage mediated by lipotoxicity and oxidative stress acts as a promoting stimulus, responsible for the release of danger signals which, in turn, activate sterile inflammatory pathways and, therefore, adaptive and native immunity components; in time, these elements amplify tissue damage and contribute to NAFLD progression [128]. Intrinsically, this process is orchestrated by multiple factors such as pro-inflammatory cytokines, resident or recruited immune cells, and even proteins generated within the hepatic tissue known as hepatokines [129]. Kupffer cells (KCs) in particular have been demonstrated to be the main source of hepatic inflammation. The release of damage-associated molecular patterns (DAMPs) leads to the activation of pattern recognition receptors (PRRs)—among them, Toll-like-receptors (TLRs). These receptors transmit triggering signals to stimulate the conversion of KCs into a pro-inflammatory phenotype able to promote chemokine and cytokine secretion as well as the exacerbation of immune cell activation in the liver [130]. In addition, the role of hepatic stellate cells (HSCs) is essential to extracellular matrix (ECM) renovation and, consequently, fibrosis and cirrhosis development through a phenotypic switch promoted by tissue damage responsible for the transformation of HSCs into myofibroblast-like cells [131].

**Figure 2:**
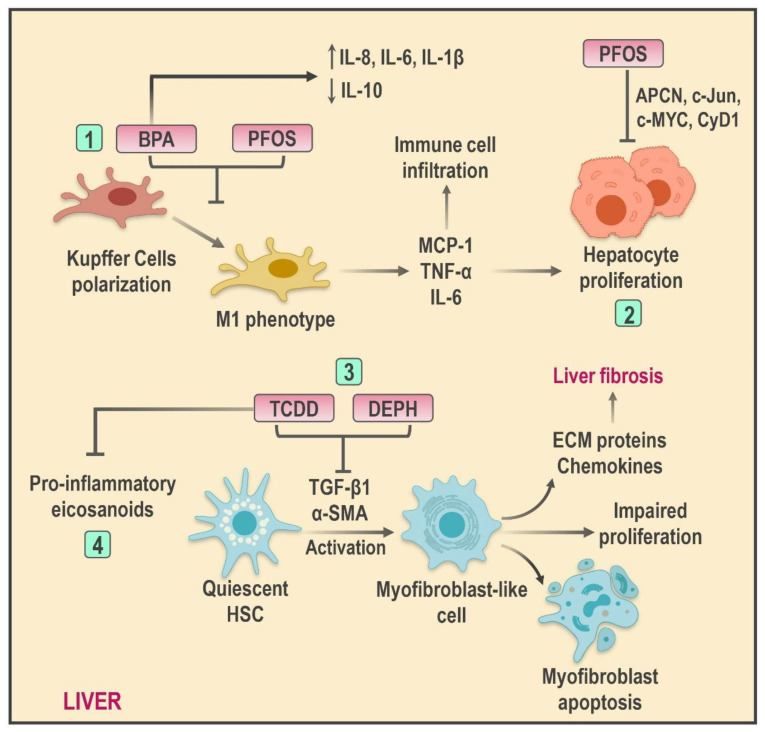
Schematic illustration of the effects of EDCs on hepatic inflammation and non-alcoholic steatohepatitis (NASH) progression. Several EDCs promote hepatic inflammation through diverse mechanisms, such as (1) induction of cytokine production and Kupffer cell polarization to a pro-inflammatory phenotype; (2) increased hepatocyte proliferation and immune cell infiltration; (3) HSC transformation to myofibroblast-like cell by promoting an imbalance between proliferation/apoptosis and, consequently, liver damage and fibrosis development; (4) increased pro-inflammatory eicosanoids production. Abbreviations: BPA: Bisphenol A; PFOS: Perfluorooctane sulfonate; DEPH: Di-(2-ethylhexyl) phthalate; TCDD: 2,3,7,8-tetrachlorodibenzo-*p*-dioxin; HSC: Hepatic stellate cell; ECM: Extracellular matrix; MCP-1: Monocyte chemoattractant protein-1; α-SMA: alpha-smooth muscle actin; TGF-β1: Transforming growth factor beta-1.

Given this hypothesis, it is plausible to consider EDC-mediated hepatotoxicity as a significant inducer of hepatic inflammatory responses (Figure 2). Similarly, the direct impact of these substances in NASH development through pro-inflammatory microenvironment promotion has been described [109,132]. In this context, recent in vitro studies carried out in HepG2 cells have reported the active participation of BPA in hepatic inflammation through the release of pro-inflammatory cytokines such as IL-8 and TNF-α [124]. In a similar way, Acaroz et al. reported increased levels of TNF-α, IL-6, and IL-1β as well as IL-10 reduction after low oral BPA dose (25 mg × kg^−1^) exposition; furthermore, these modifications promote the development of pro-inflammatory microenvironments along with dose-dependent histopathological changes in the liver of Wistar rats [133].

The pro-inflammatory effect of BPA on the liver cannot be exclusively attributed to cytokine secretion. EDC exposure has turned out to be a key factor in the activation of KCs within the liver due to the polarization of these cells into an M1 phenotype (M1KC) as well to the augmented production of monocyte chemoattractant protein-1 (MCP-1) and pro-inflammatory cytokines in C57BL/6J mice-isolated KCs. Experimental studies have demonstrated the effective blockage of M1KC differentiation through the usage of the estrogenic antagonist ICI 182780; therefore, ER signaling has been proposed as a potential linking mechanism between BPA and this event [134]. Likewise, PFOS exposition exerts a significant effect in KC activation with the subsequent release of TNF-α and IL-6 in a JNK and NF-κB activation-dependent mechanism. In addition, increments in these cytokines promote *apcn*, *c-jun*, *c-myc*, and *cyd1* increments and, in consequence, hepatocyte proliferation. These findings suggest that PFOS-induced KC activation plays a major role in HCC development [135].

On the other hand, DEPH has been associated with hepatic fibrosis and pro-inflammatory phenomena as a result of observable increments in steatosis, hepatocyte necrosis, and immune infiltration after TNF-α and IL-6 increments following different doses of DEPH in addition to increased levels of profibrogenic factors such as α-SMA, COL-I, COL-III, and TGF-β1 in LX-2 cells [136]. In this regard, TGF-β1 turns HSCs into a DEPH-susceptible population [137]. Recently, Lee et al. [138] pointed out alterations in cholesterol metabolism produced by low doses of DEPH, which elevates the synthesis of endogenous cholesterol in HSCs and proliferation/apoptosis imbalances. These events could be linked to their phenotypical transformation, accelerating liver damage and fibrosis in murine models. The effect of DEPH over the ECM could be stimulus-dependent since increased collagen production has been registered following the administration of carbon tetrachloride (CCL_4_).

2,3,7,8-tetrachlorodibenzo-*p*-dioxin (TCDD) is another EDC able to induce HSC activation since its exposure seems to promote HSC transition to myofibroblasts and increased proliferation rates through a PI3K-dependent mechanism. Interestingly, increments in HSC activity markers such as α-SMA and MCP-1 have been registered despite PI3K inhibition; this observation suggests that TCDD might mediate HSC activation by binding directly to AhR in the cellular nucleus [139]. Finally, the role of TCDD in hepatic inflammation has been attributed to its influence in lipid mediators derived from omega-6 polyunsaturated fatty acids (ω-6 PUFAs). Increased production of pro-inflammatory eicosanoids derived from lipoxygenase and CYP450 pathways such as LTB_4_ and LTB_3_ following AhR activation has been documented. In this sense, TCDD effects on the ω-6/ω-3 PUFA ratio represent a potential mediator mechanism in steatohepatitis progression [140].

### 4.4. Epigenetic Changes and Transgenerational Inheritance

Current evidence proposes alternative mechanisms to nuclear receptor signaling or mitochondrial dysfunction for NAFLD development in response to EDCs. Epigenetic changes have been pointed out as a crucial factor in NAFLD pathogenesis. Epigenetic changes are genetic expression re-editing processes that occur in the absence of nucleotide sequence alterations; these changes include processes such as DNA methylation and histone modifications that could lead to the development of phenotypical variations [141]. During NAFLD, EDC exposure is associated with modifications in histones and DNA methylation patterns, which, in turn, produce alterations in the adjustment profile of the liver. Adjustments occur due to variations in methyl donor availability, histone methyltransferase modifications, changes in dioxygenase activities, imprint imbalances, non-coding RNA alterations, and phosphate-dependent signaling pathways’ activation [142].

Epigenetic reprogramming generally remains transcriptionally repressed until environmental stimuli trigger its activation [143]. In this sense, the transmission of new adjustments into the progeny will take place if the reprogramming is carried out in germinal instead of somatic cells [144,145]. The occurrence of transgenerational events depends on fetal exposure to EDCs, and the effects can be identified in the F3 generation. Once the expectant (F0) is exposed to toxic agents, the fetus germinal line (F1) will be exposed as well. Afterward, the F1 cells will engender the F2 generation, and therefore, the F3 will be the only generation indirectly exposed to the teratogen. Thus, the mechanism responsible for the phenotypical manifestation in this generation is the epimutation transferred by the ancestral germinal line (F0) [24,146].

Certain chemical agents such as BPA are linked to these effects. Despite its short lifespan in human adults, the metabolic rates are considerably slower in fetuses and neonates [147]. Hence, EDC exposure in early life is considered more harmful compared to adulthood [148]. Studies have shown the existence of hypermethylation/hypomethylation in cytosine-phosphate-guanine (CpG) sequences located near to primers, histone modifications, and modulation of non-coding RNA, including microRNA after neonatal and uterine BPA exposure [149,150,151,152,153,154].

Among the first pieces of evidence, Ma et al. reported that BPA oral exposure (50 μg/kg/day) during nursing and gestation in Wistar rats induced IR, glucose intolerance, and increased body weight in the 21st week of pregnancy by promoting hepatic DNA global hypomethylation as well as glucokinase (*gck*) gene hypermethylation, which codifies a glycolysis rate-limiting enzyme, essential in hepatocyte glucose utilization. Furthermore, despite the absence of histopathologic changes, DNA hypomethylation and *gck* reduced expression induced by BPA could represent a potential key mechanism in hepatic IR development and T2DM preceding NAFLD [155].

Moreover, chronic exposure (10 months) to BPA leads to significant increments in hepatic cholesterol and TG levels in male mice as a result of *srebf1* and *srebf2* hypomethylation and its consequent augmented expression. Additionally, decreased mRNA and DNA methyltransferase levels have been documented. The stimulating effects of DNA hypomethylation on hepatic lipid accumulation establish epigenetic reprogramming as an important mechanism in NAFLD development [156].

Similarly, recent studies suggest that epigenetic mechanisms might represent a key pathway to explain the effects of metalloids in human health. In this regard, the influence of arsenic in epigenetic modifications has been pointed out, considering the global hypomethylation triggered in the liver of male mice after chronic exposure to the metal as well as the hypomethylation observed in ERα primers, linked to increased cellular hypertrophy and hepatic steatosis [157]. Furthermore, intrauterine exposure to trivalent arsenic (As^III^) can increase NAFLD risk and cardiometabolic diseases in murine models. Increased liver weight and TG levels were observed after 13 weeks as a consequence of tricarboxylic acid cycle (TCA) deterioration and consequential increments in FFA synthesis [158].

Differently, DEPH exposure in utero favors the development of metabolic disorders in murine descendants and considerable elevations in visceral adiposity, and *tbx15* and *gpc*4 have also been observed in F1 generations, presumably as a result of modifications in DNA methylation. Increased gene expression is correlated with metabolism disruption in adipose tissue and, therefore, predisposition to NAFLD development [159].

Comparably, exposure to different plastic-derived products (BPA, DEHP, and DBP) in various doses promotes transgenerational epigenetic inheritance at the beginning of the descendant’s adult life by stimulating obesity development and other disorders that will mainly affect body weight and visceral adiposity in the F3 generation [160]. Additionally, dichlorodiphenyltrichloroethane (DDT) has shown a significant effect in the promotion of this mechanism. Transitory exposure to DDT in rats increased obesity incidence and other associated pathologies in males and females of the F3 lineage [161]. Lastly, despite evidence concerning transgenerational epigenetic inheritance in adiposity and lipid metabolism, further investigations are necessary to link this mechanism to EDCs and NAFLD exposure.

## 5. Endocrine-Disrupting Chemicals and NAFLD: Clinical Evidence

Considering the exposure of the molecular mechanism associated with EDCs in NAFLD pathogenesis, it is necessary to ponder the existing clinical–epidemiological relationship between these elements. In recent years, this topic has proven to be a challenge. An extensive range of experimental studies performed in animal models with different BPA [17,98,162], phthalates [94,163], TBT [164,165], dioxins [166,167,168], DDE [169], PFOA [170], and PFOS [90,171] doses have been conducted. Nevertheless, research of EDCs’ implications in clinical studies has been unsatisfactory; as a result, causal inferences in such investigations remain unknown, mainly due to the methodological challenges that comparisons between exposed and non-exposed populations present.

Epidemiological studies (Table 2) have reported positive associations between POPs, pathogenic elements, and NAFLD serum markers. In this sense, a transversal cohort study was conducted in 436 individuals, showing increased ALT levels positively associated with 20 different kinds of PCBs [172]. Similarly, Kim et al. found that PCB serum levels were correlated with elevated concentrations of AST, ALT, GGT, TG, and TCHO in obese individuals [173]. On the other hand, Pazderova-Vejlupkova et al. conducted a longitudinal study with 55 male individuals, dedicated to TCDD production; during the 10-year exposure, the participants developed TCDD chronic toxicity. Researchers reported metabolic disorders in approximately half of the participants, dyslipidemia being the most common alteration; simultaneously, a third of the intoxicated individuals presented altered hepatic function parameters that suggested moderated hepatic damage. Likewise, findings such as KCs’ activation, periportal fibrosis, and mild steatosis were described after histological examination of samples obtained via necropsies and/or biopsies [174].

Furthermore, a transversal study exposed 55 participants to polychlorinated dibenzo-*p*-dioxins and dibenzofurans (PCDD/Fs). Hepatic parameters and POPs determined after the exposure revealed associations between elevated PCDD/Fs levels and fatty liver as well as increased GGT in individuals with high BMI, therefore suggesting that dioxin exposure affects fatty liver prevalence among exposed subjects [175]. Likewise, a clinical trial carried out in 6–10-year-old children exposed to TCDD following the Seveso, Italy, accident, showed altered GGT and ALT levels in children exposed to POPs, indicating potential hepatic dysfunction [176].

Perfluoroalkyl substances (PFASs) such as PFOA and PFOS are other POPs associated with NAFLD in children as well as adults. Moreover, a trial including 2216 adults from the National Health and Nutrition Examination Survey (NHANES) database assessed the relationship between PFOA plasmatic levels and hepatic enzyme concentrations, reporting that for every PFOA concentration unit increase, ALT and GGT serum levels increased by 1.86 and 0.08 units, respectively. Furthermore, the association between PFOA and hepatic parameters was significant among metabolic syndrome, insulin-resistant, and/or obese individuals [177]. Similar results were observed in a study that evaluated perfluorinated chemicals and various hepatic function markers in individuals. Using the NHANES database, an evident correlation between PFOA and ALT, GGT, and bilirubin levels was observed. Furthermore, every PFOS quartile was associated with proportionally elevated levels of bilirubin [178]. Additionally, a different trial conducted in 74 children diagnosed with NAFLD suggested that PFAS exposure could play a significant role in NAFLD progression based on the results of histologic liver tests and PFOS/PFOA plasma level determination. In fact, POP elevations were correlated with worsening of the disease [179].

Clinical associations between non-persistent EDCs such as BPA and phthalates with NAFLD have been explored over recent decades. A study carried out by Lang et al. reported that higher BPA concentrations are linked to clinically abnormal GGT (Odds Ratio (OR) 1.29; 95% Confidence Interval (CI), 1.14–1.46; *p* < 0.001) and ALP (OR 1.48; 95% CI, 1.18–1.85; *p* = 0.002) concentrations in adults [180]. Moreover, higher urinary BPA concentrations have been associated with augmented hepatic dysfunction in elderly subjects (OR 2.66; 95% CI: 1.15–5.90) due to significant links between urinary BPA, AST, ALT, and GGT [181]. Tarantino et al. [182] reported high, weight-independent BPA levels in women with polycystic ovary syndrome, associated with elevated AST, ALT, and GGT levels and hepatic steatosis. In parallel, Khalil et al. [183] performed a transversal study where the correlation between reduced urinary BPA levels and age reductions in male obese children (*n* = 17) was observed. These findings were associated to elevated serum activity of AST.

Finally, phthalates exposure has been associated with hepatic dysfunction and NAFLD. In this sense, a transversal measuring study monoethyl phthalate (MEP) urinary levels, mono-(2-ethylhexyl) phthalate (MEHP) and serum levels of hepatic parameters were determined in 305 volunteers with normal body weight, overweight, or diabetes. Among individuals with a regular body mass and MEP^+^, ALT and AST levels were significantly higher; in contrast, MEHP^+^ levels were correlated with GGT exclusively. Moreover, participants with normal body weight had a negative correlation between MEP, TCHO levels, and LDL-c, while in obese individuals, MEP levels were associated with elevated levels of ALT, AST, and TG. Furthermore, in diabetic patients, MEP concentrations were correlated with GGT levels. According to these results, deterioration of hepatic function could be associated with ubiquitous exposure to phthalates [184]. Similarly, a study aimed to assess MEP and MEHP effects on the hepatic function and lipid metabolism of 102 males and described the association between phthalates exposure and dramatic ALT and AST increments in plasma, as well as their correlation with hypertriglyceridemia and HDL-c reductions [185].

Lastly, numerous indexes such as the fatty liver index (FLI), the triglyceride and glucose index (TyG), homeostatic model assessment (HOMA), visceral adipose index (VAI), lipid accumulation product (LAP), body mass index (BMI), and waist circumference (WC) have been studied as potential NAFLD predictors [186,187,188,189,190]. However, published evidence regarding the association between these indicators and EDCs is very limited. In this context, a cross-sectional trial by Hatch et al. reported associations between increased levels of phthalates and anthropometric indexes such as BMI and WC [191]. Similarly, the study carried out by Stahlut et al. exposed a significant association between EDCs and increased WC and HOMA indexes [192]. Similar findings were found in investigations performed by Dee Geiger et al., La Merril et al., and Lee et al. in which increased values of HOMA, WC, and BMI were linked to multiple EDCs [193,194,195]. Nevertheless, further studies with robust methodologies capable of assessing the relationship between these metabolic indexes and EDC exposure levels are required.

## 6. Conclusions

NAFLD is the most common hepatic disease; it has rapidly become a major epidemiological problem, projecting itself as the main hepatic transplant indication for 2030 [196]. Within the complex spectrum of its pathophysiology, environmental exposure to chemical substances present in wildlife and industrial spaces such as EDCs represents a relevant trigger.

EDCs’ effects in NALFD occur at the expense of NR interactions, activating transcriptional factors which, in turn, trigger imbalances between lipid influx/efflux in the liver, promote mitochondrial dysfunction, and boost key inflammatory responses in NASH progression. Furthermore, the relevance of the exposures depends on time and life cycles, with early exposure being the most susceptible period for DNA and histone modifications, which increases the risks for NAFLD development in adult life. In this context, the exponential role of EDCs in the pathogeny of the disease is undeniable. Nevertheless, the evidence in human trials remains scarce; therefore, further research with larger samples, more specific side effects, and longer follow-up periods aiming to clarify the exposure to these substances is required.

## Figures and Tables

**Table 1:** Origin-based classification of main endocrine-disrupting chemicals (EDCs).

Type	Chemical Name	Abbreviation	Introduction Date	Restricted/ Banned	Source
**Residential**					
Phenols	Bisphenol A Bisphenol S	BPA BPS	1960	Restricted	Polycarbonate plastics, epoxy resins, plastic toys and bottles, lining of food cans
Phthalates	Mono-(2-ethylhexyl)-phthalate Di-(2-ethylhexyl)-phthalate Dibutyl-phthalate Dicyclohexyl phthalate	MEHP DEHP DBP DCHP	1920	Restricted	PVC: lubricants, perfumes, cosmetics, medical tubing, wood finishes, adhesives, paints, toys, emulsifiers in food, flooring, personal care products
Perfluorinated chemicals	Perfluorooctanoic acid Perfluoroctanesulfonates Perfluorononanoic acid Perfluorohexanesulfonic Acid	PFOA PFOS PFNA PFHxS	1940	Restricted	Contaminated food and water, dust, floor waxes, firefighting foam, electrical wiring, lining of food wrappers, stain resistant carpeting
**Industrialist**					
Dioxins	Polychlorinated Dibenzo P	PCDD	1872	Restricted	By-product of chlorinated herbicide production, smelting, chlorine bleaching of paper
Polychlorinated biphenyls	Polychlorinated biphenyls Polybrominated biphenyls Polychlorinated terphenyls Polychlorinated naphthalenes	PCBs PBBs PCTs PCNs	1927	Banned	Contaminated air and food, skin contact with old electrical equipment
Polycyclic aromatic hydrocarbons	Benzo[*a*]pyrene, anthracene, acenaphtylene, fluorene	PAH	–	Restricted	Products of fuel burning
Alkylphenols	Nonylphenol Octylphenol	NP OP	–	Restricted and banned in certain areas of use in the USA	Surfactants, detergents, emulsifiers; fish, drinking water, personal care products
Heavy metals	Arsenic	As	–	Restricted	Pesticides, smelting, industrial waste, drinking water, soil, seafood, rice, mushrooms, poultry
	Mercury	Hg	–	Restricted	Mining, waste incineration, manufacturing; fish, shellfish, medical/dental procedures
	Cadmium	Cd	–	Restricted	Soil, water, air; leafy vegetables, peanuts, soybeans, sunflower seeds; inhalation products of mining, combustion, waste incineration
**Agricultural**					
Dicarboximide	Vinclozolin	Vnz	1981	Banned	Diet and occupational
Organotins	Tributyltin oxide Triphenyltin	TBT TPT	–	Banned by many countries	Used as a biocide (fungicide and molluscicide), especially as a wood preservative
Organochloride	Dichlorodiphenyltrichloroethane Dichlorodiphenyldichloroethylene	DDT DDE	1940	Banned	Contaminated water, soil crops, fish, pesticides
Chlorotriazine	Atrazine	ATR	1959	Banned	Pesticide/herbicide, contaminated water and soil
**Pharmaceutics**					
Parabens	Butylparaben, methylparaben, ethylparaben, propylparaben, benzylparaben	Parabens	1924	Restricted	Antimicrobial agents for the preservation of food, paper products, and pharmaceutical products
Non-steroidal synthetic estrogen	Diethylstilbestro	DES	1941–1947	Restricted	Pharmaceutical

**Table 2:** Summary of the clinical evidence regarding endocrine-disrupting chemicals and non-alcoholic fatty liver disease.

Author [Ref]	EDC	Methodology	Results
Cave et al. [153]	PCBs	Cross-sectional cohort study evaluating the influence of environmental pollutants in serum ALT in 436 adults.	20 PCBs were positively associated with subjects that had elevated ALT levels (*p* ≤ 0.05).
Lee et al. [156]	Dioxins	Cross-sectional study which evaluated the associations between serum PCDD/Fs levels and adverse hepatic-related health outcomes in adults.	In comparison to the control group, the risk of fatty liver increased significantly in adults with higher BMI and higher serum PCDD/Fs (OR = 27.00, 95% CI = 4.47–229.58).
Jin et al. [160]	PFAS	Cross-sectional study assessing the relationship of PFAS to histologic severity of NAFLD in 74 children.	The odds of having NASH significantly increased with the increase in plasma concentrations of PFOS (OR: 3.32, 95% CI: 1.40–7.87), PFHxS (OR: 4.18, 95% CI: 1.64–10.7), and PFAS composite variable (OR: 4.89, 95% CI: 1.86–12.8).
Lin et al. [158]	PFOA	Cross-sectional cohort study examining the relationship between serum levels of PFOA and the levels of liver enzymes in 2216 adults.	When PFOA concentration increased by one unit, the serum levels of ALT and GGT increased by 1.86 (95% CI, 1.24–2.48; *p* = 0.005) and 0.08 units (95% CI, 0.05–0.11; *p* = 0.019), respectively.
Tarantino et al. [163]	BPA	Cross-sectional study that evaluated the effects of increased serum BPA levels on low-grade chronic inflammation and hepatic steatosis in women with polycystic ovary syndrome.	Higher serum levels of BPA were associated with higher grades of hepatic steatosis and AST, ALT, and GGT (*p* ≤ 0.05).
Milošević et al. [166]	Phthalates	Cross-sectional study with 102 male participants assessing the influence of MEP and MEHP on the liver function and cardiometabolic risk factors.	MEP+ normal weight group had statistically significant elevated transaminase serum levels. Moreover, there were correlations found between MEP concentration in urine samples and TAG serum levels (r^2^ = 0.33; *p* < 0.01), VAI (r^2^ = 0.41; *p* < 0.01), LAP (r^2^ = 0.32; *p* < 0.01), and TAG-to-HDL ratio (r^2^ = 0.40, *p* < 0.01) among obese subjects.

**Abbreviations:** OR: Odds ratio; CI: Confidence interval; NAFLD: Non-alcoholic fatty liver disease; PCB: Polychlorinated biphenyl; ALT: Alanine aminotransferase; PCDD/Fs: Polychlorinated dibenzo-p-dioxins and dibenzofurans; BMI: Body mass index; PFAS: Perfluoroalkyl substances; NASH: Non-alcoholic steatohepatitis; PFOS: Perfluorooctane sulfonate; PFHxS: Perfluorohexane sulfonic acid; PFOA: Perfluorooctanoic acid; GGT: Gamma-glutamyl transferase; BPA: Bisphenol A; AST: Aspartate aminotransferase; MEP: Monoethyl phthalate; MEHP: Mono-(2-ethylhexyl) phthalate; VAI: Visceral adiposity index; LAP: Lipid accumulation product; HDL: High-density lipoprotein; TAG: Triacylglyceride.
